# Computer-vision-based technology for fast, accurate and cost effective diagnosis of malaria

**DOI:** 10.1186/s12936-015-1060-1

**Published:** 2015-12-30

**Authors:** Bina Srivastava, Anupkumar R. Anvikar, Susanta K. Ghosh, Neelima Mishra, Navin Kumar, Arnon Houri-Yafin, Joseph Joel Pollak, Seth J. Salpeter, Neena Valecha

**Affiliations:** National Institute of Malaria Research, Sector 8 Dwarka, New Delhi, 110 077 India; National Institute of Malaria Research Field Unit, Bengaluru, India; Sight Diagnostics, 1 Agudat Hasport Hapoel, Jerusalem Technology Park, Jerusalem, Israel

**Keywords:** Malaria, Diagnosis, PCR, Device, Sensitivity, Specificity

## Abstract

**Background:**

Microscopy has long been considered to be the gold standard for diagnosis of malaria despite the introduction of newer assays. However, it has many challenges like requirement of trained microscopists and logistic issues. A vision based device that can diagnose malaria, provide speciation and estimate parasitaemia was evaluated.

**Methods:**

The device was evaluated using samples from 431 consented patients, 361 of which were initially screened by RDT and microscopy and later analysed by PCR. It was a prospective, non-randomized, blinded trial. Quantification of parasitaemia was performed by two experienced technicians. Samples were subjected to diagnosis by Sight Dx digital imaging scanning.

**Results:**

The sensitivity and specificity of the SightDx P1 device for analysed samples were found to be 97.05 and 96.33 %, respectively, when compared to PCR. When compared to microscopy, sensitivity and specificity were found to be 94.4 and 95.6 %, respectively. The device was able to speciate 73.3 % of the PCR *Plasmodium falciparum* positive samples and 91.4 % of PCR *Plasmodium vivax* positive samples.

**Conclusion:**

The ability of the device to detect parasitaemia as compared with microscopy, was within 50 % in 71.3 % of cases and demonstrated a correlation coefficient of 0.89.

## Background

According to the World Health Organization (WHO) estimates, there were ~207 million cases and 627,000 deaths due to malaria in 2012 [1]. Half of the world’s population is at risk of being infected by malaria. Definitive diagnosis of malaria is imperative for rapid treatment, reducing morbidity and mortality, preventing unnecessary use of anti-malarials and increased drug resistance [2]. As a result, the WHO now recommends that all cases of suspected malaria should be confirmed using malaria diagnostic tests prior to treatment. Microscopy continues to be the gold standard for diagnosis of malaria [2] but is time and labour intensive, requires qualified personnel, and is subjective. Rapid diagnostic tests (RDTs) have revolutionized the diagnosis of malaria, particularly in remote areas [3] but suffer from difficulties in transport and storage, persistence of Hrp-2 in the blood, poor detection at very low parasitaemia, and inability to quantify the parasites. Other techniques available for diagnosis include immunologic techniques, Polymerase Chain Reaction (PCR), Loop-mediated isothermal amplification (LAMP), all of which present unique challenges. PCR has potential to detect low parasitaemia but is expensive, time consuming, and requires highly trained personnel. LAMP is being evaluated [4, 5] and is still in early stages of development [6].

The SightDx P1 Device aims to overcome these deficits: the computer-vision-based technology is designed for fast, accurate and cost effective diagnosis of malaria in blood samples. Additionally the device is able to rapidly report parasitaemia levels after scanning over 1 million red blood cells.

## Methods

This trial aimed to evaluate the efficacy of the device, as measured by the sensitivity and specificity at different levels of parasitaemia. The device was evaluated on 431 consented patients. Sensitivity and specificity of the device were compared against PCR and microscopy. Rapid diagnostic tests (RDTs) were used for initial patient screening and were also compared against ground truth. All patients were symptomatic. The primary endpoint was to assess the sensitivity and specificity of the device using PCR as the gold standard. The secondary endpoints were species identification, accuracy of parasitaemia estimation and comparison with microscopy.

### Study design

This was a single center, prospective, non-randomized, blinded trial. All technicians were blinded to the results of each branch of the study. Comparison between study outcomes was done only after data collection and the examination was complete.

### Study procedures

All patients presenting with fever at the NIMR clinic were considered eligible for enrollment and were given the option to participate. Informed consent was obtained prior to sample collection. Suspected patients for *Plasmodium vivax* and *Plasmodium falciparum* were routinely diagnosed in the malaria clinic of Wenlock Hospital, Mangalore, by examining Giemsa-stained slides (and/or malaria RDTs as routinely used in the clinic; SD Malaria Ag Pf/Pv in this case). Malaria treatment was solely based on the malaria clinic diagnosis process and patients’ course of treatment [7] was not changed due to the Parasite P1 device or by the NIMR microscopy diagnoses. In parallel, about 4 µL of blood was collected in microfuge tube containing EDTA. These samples were scanned onsite by using the P1 device, while P1 final analysis was conducted blindly in SightDx’s R&D facility in Israel. In addition, blood was also collected on filter paper spots for PCR evaluation at NIMR, and was used for the preparation of two Giemsa-stained smears for microscopic examination at NIMR. The Giemsa-stained smears and filter papers were transported to NIMR, New Delhi.

The Giemsa slides were reviewed by a NIMR microscopist at Wenlock NIMR clinic in Mangalore, and by a second microscopist at the NIMR laboratory in New Delhi. If there was disagreement between the two reads, or a discrepancy in parasitaemia level of greater than 50 %, a third microscopist reviewed the slide and made a final determination.

Diagnostic PCR assays were carried out at NIMR. For this, DNA was isolated from filter paper using the QIAamp mini kit (51306) according to the manufacturer’s instructions and stored at −20 °C until PCR could be completed. Nested PCR was performed with primers described previously [7, 8]. Known positive sample from parasite bank of NIMR and previously uninfected individual’s blood was used as positive and negative control, respectively.

The SightDx digital imaging scanning was performed onsite. A blood droplet was stained and diluted in a proprietary solution. This diluted sample was placed inside a flow-cell disposable slide. The slide was then loaded into the P1 machine where it was scanned automatically. Each field was autofocused and stained covering a total of 270 fields corresponding to 0.2 μl of blood. The complete scan took less than 5 min per sample. At peak participant volume, 56 patients were analysed in an 8 h working day.

Computer vision and statistical models were used to detect the malaria parasites. The algorithm used fluorescent cues to detect hotspots and then classified these into white blood cells, parasites, or “other”. The algorithm also estimated RBC density. Using statistical models, SightDx P1 determined infection status, parasitaemia levels, and species. Diagnosis and parasitaemia are statistical constructs, and there is an *Internal Calibration Parameter* that determines whether a sample is considered “negative” that can be adjusted to optimize the tradeoff between sensitivity and specificity for the use at hand (e.g. screening or confirmation).

### Statistical methods

Sensitivity and specificity analysis and 95 % confidence intervals (CIs) were computed using a 2 × 2 table for outcomes of the tested device and the reference outcome. Test sensitivity (conditional probability that the test is positive if the condition is positive), calculated by the following formula:

$${{{\text{Sensitivity}} = \left( {{\text{True}}\;{\text{Positive}}} \right)} \mathord{\left/ {\vphantom {{{\text{Sensitivity}} = \left( {{\text{True}}\;{\text{Positive}}} \right)} {\left( {{\text{True}}\;{\text{Positive}} + {\text{False}}\;{\text{Negative}}} \right) \times 100}}} \right. \kern-0pt} {\left( {{\text{True}}\;{\text{Positive}} + {\text{False}}\;{\text{Negative}}} \right) \times 100}}$$

Test specificity (conditional probability that the test is normal if the condition is normal (negative), calculated by the following formula:

$${{{\text{Specificity}} = \left( {{\text{True}}\;{\text{Negative}}} \right)} \mathord{\left/ {\vphantom {{{\text{Specificity}} = \left( {{\text{True}}\;{\text{Negative}}} \right)} {\left( {{\text{True}}\;{\text{Negative}} + {\text{False}}\;{\text{Positive}}} \right) \times 100}}} \right. \kern-0pt} {\left( {{\text{True}}\;{\text{Negative}} + {\text{False}}\;{\text{Positive}}} \right) \times 100}}$$

Kappa Coefficients were calculated for analysing the agreement between the diagnoses of the two microscopists. The data was analysed using the SAS ^®^ version 9.1 (SAS Institute, Cary North Carolina).

## Results

**Fig. 1:**
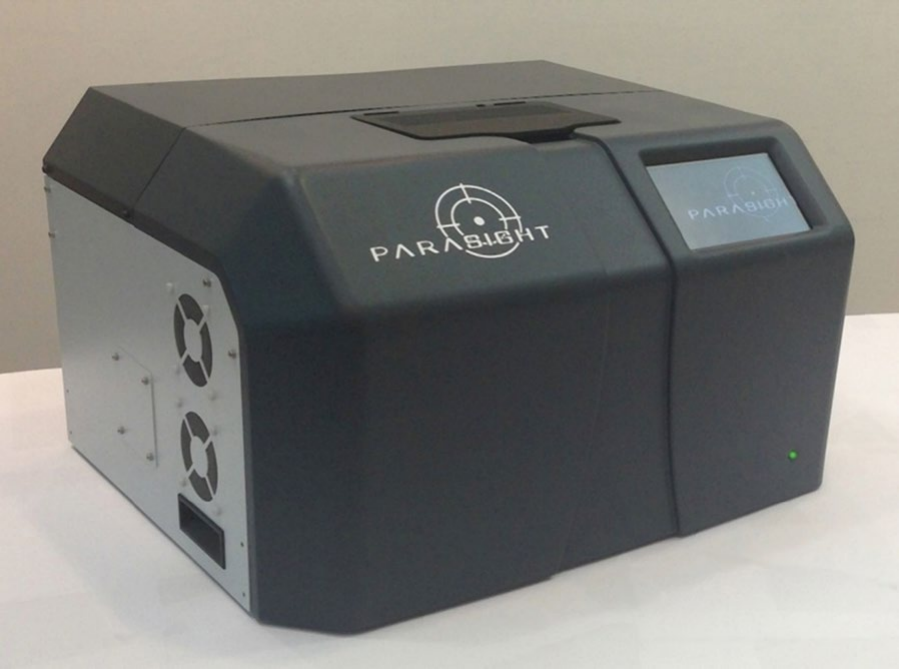
The SightDx P1 Device. A compact platform for malaria diagnostics

The SightDx P1 device is a tabletop device for malaria diagnostics (Fig. 1). Cartridges are placed in the device, scanned and output is presented on the device touch screen.

**Fig. 2:**
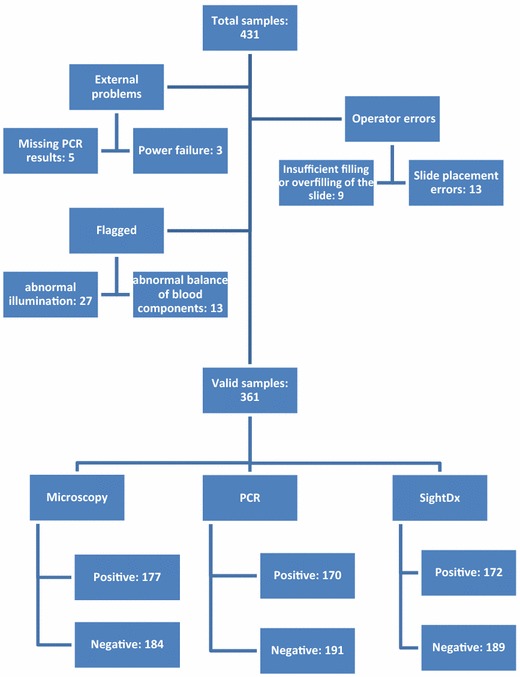
Description of the samples. A total of 431 samples were collected and 361 were analyzed. Removed samples were due to technical errors or due to flagged samples

The evaluation of SightDx P1 device performance was based on 431 febrile patients reporting to the Malaria Clinic at Wenlock Hospital, Mangalore and consenting to participate. Figure 2 shows the description of the evaluable samples. Of 431 patients consented to participate, 70 samples were not included in the analysis for various reasons: operator errors, such as slide placement error, insufficient filling or overfilling of the slide (n = 22), external technical problems, such as interruptions during running of the test and power failure (n = 3), missing PCR results (n = 5) and samples marked as undecided by the automated algorithm (n = 40), which need further investigation. These 40 samples will henceforth be referred to as ‘flagged’ samples in this article. Thus, the study’s population involved 361 samples that were evaluated by SightDx device, PCR and microscopy.

### SightDx compared to PCR and microscopy

The device’s sensitivity and specificity, when compared to PCR, were found to be 97.05 % (95 % CI 94.5–99.6) and 96.33 % (95 % CI 93.7–99.0), respectively.

SightDx sensitivity and specificity compared to microscopy were found to be 94.4 % (95 % CI 90.9–97.8) and 95.6 % (95 % CI 92.7–98.6), respectively (Table 1) .

**Table 1:** Sensitivity and specificity of P1 device versus PCR and microscopy

Comparator	Sensitivity			Specificity		
	Percent	n/N	95 % CI	Percent	n/N	95 % CI
Microscopy	94.4	167/177	90.9–97.8	95.6	176/184	92.7–98.6
*PCR*	97.05	165/170	94.5–99.6	96.33	184/191	93.7–99.0

### RDT compared to PCR and microscopy

The RDT’s sensitivity and specificity, when compared to PCR, were found to be 93.5 % (95 % CI 89.8–97.2) and 96.3 % (95 % CI 93.7–99.0), respectively. Sensitivity and specificity of the RDT compared to microscopy were found to be 94.3 % (95 % CI 90.0–97.8) and 96.7 % (95 % CI 94.2–99.3), respectively (Table 2).

**Table 2:** Sensitivity and specificity of RDT versus PCR and microscopy

Comparator	Sensitivity			Specificity		
	Percent	n/N	95 % CI	Percent	n/N	95 % CI
Microscopy	94.3	167/177	90.9–97.8	96.7	178/184	94.2–99.3
*PCR*	93.5	159/170	89.8–97.2	96.3	184/191	93.7–99.0

### Speciation

The ability of the device to identify two types of malaria species was compared with PCR. The current version of the device did not support mixed infection reporting. From samples found to be positive by both PCR and SightDx, SightDx speciated 73.3 % of PCR *P. falciparum* + samples and 91.4 % of PCR *P. vivax* + samples, as shown in Table 3. Notably, all of the mixed infection samples were positively detected by SightDx as infected. Eleven mixed infections were reported as *P. vivax*, two were reported as *P. falciparum*, while one was reported positive.

**Table 3:** Speciation accuracy divided according to treatment groups

	SightDx accuracy (%)	In numbers	95 % CI
*P. vivax*	91.4	96/105	86.1–96.8
*P. falciparum*	73.3	34/46	61.2–86.6
Mixed infection	0	0/14	NA

### Parasitaemia

**Fig. 3:**
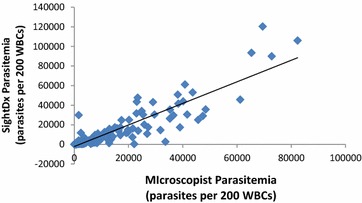
Correlation between Sight Dx parasitaemia and Microscopy parasitaemia. The microscopist counted 200WBCs

To correlate the microscopists analysis with the parasitaemia provided by the SightDx device Pearsons correlation and Spearmans correlation analysis was carried out. Spearman’s rank correlation coefficient analysis is preferred for monotonic relationships. The Spearman’s rank correlation coefficient and the Pearsons correlation coefficient calculated when comparing SightDx’s parasitaemia reporting to microscopy on infected samples. Following the WHO criteria, SightDx was analysed on whether or not it produced parasitaemia levels of ±50 % of microscopy. Microscopy parasitaemia was considered as the average of the two microscopists’ estimation, or the third microscopist’s estimation in cases of disagreement. SightDx was within 50 % in 71.3 % of cases (in 117 out of 164). The overall correlation between the two data sets can be seen in Fig. 3.

## Discussion

The study evaluates a novel platform for diagnosing malaria. It showed sensitivity of 97.5 % and specificity of 96.33 % when compared to PCR. Moreover, the study showed that the platform provides high sensitivity in cases of low parasitaemia. Going by the WHO standard of 95 % sensitivity for malaria diagnosis, the device has excellent sensitivity. This is the first example of a complete vision based malaria diagnostic platform, as attempts to develop vision-based device have been made in the past with limited success [9–12].

The device was able to speciate 73.3 % of the *P. falciparum* positive samples and 91.4 % of *P. vivax* positive samples compared to PCR. However currently it does not detect mixed samples since a large enough database has not been collected. There is scope of improvement in this area since there are many countries having both the species and also have good significant proportion of mixed infections [13].

Enumeration of parasites in malaria patients is extremely important to know the severity of malaria, as a prognostic indicator, drug efficacy studies and clinical trials. Despite availability of various tools for malaria diagnosis, microscopy is the mainstay for quantitation of parasites. Though accurate, easy to perform, cost effective, it is labor intensive and can vary between technicians. Very few tools are able to estimate the parasitaemia in malaria patients. Real time PCR has the potential to quantify parasites and has been proved to be useful [14]. Flow cytometry could also accurately measure parasitaemia in rodent malaria [9, 10]. Nonetheless, these methods need sophisticated equipment and are not cost effective. The SightDx P1 platform has a very powerful use for quantitative estimation of parasitaemia. It could detect the parasite count within 50 % of that detected by microscopy in 71.3 % of the cases. Most notably, the device demonstrated a correlation coefficient of 0.89 demonstrating a strong statistical similarity, and convincingly showing the relevance of the technology in clinical malaria assessment.

Strikingly, the test demonstrated significantly improved sensitivity and specificity when compared to the RDT used in the study. RDTs are known to have poor sensitivity specifically at low parasitaemia. Moreover, even when positive bands do appear, many times they are weak lines, which are hard to interpret. While RDTs showed a sensitivity of only 93.5 % the P1 platform showed a sensitivity of 97.05 %. The SightDx device offers better sensitivity and specificity and clearly states whether the sample is positive or negative, preventing mis-interpretation or difficulties in analysis.

Though the device showed good sensitivity and specificity against PCR, several improvements are still needed. The software version used in this study is not equipped to detect mixed infections, and while all the mixed infection samples were diagnosed as infected by the device, it interpreted them mostly as *P. vivax*. Furthermore, the device is capable of identifying the infection stage of a sample, but verifying the accuracy of infection stage identification was not in the scope of this study. There were operator errors responsible for indeterminate results. Such errors could be eliminated by including a modified sample-loading mechanism and improved sample-preparation procedures. The operator errors can be identified during the scanning of the sample, in which case, the user can be notified to re-run the sample. There were external conditions that caused test failures. The device could be improved to identify that an interruption has occurred and request the user to re-run the sample.

Some samples could not be analysed due to the reasons discussed in Fig. 2. However, several software and hardware correction steps were implemented since the data collection completion, aiming at dramatically lowering the rejection and undetermined sample rate. These steps include sample and slide preparation steps to reduce overfilling or insufficient slide filling and to improve staining solution consistency, slide holding improvements to reduce slide misplacement, software improvements in handling sample deviation and support for real-time user re-scan report flag.

Among the 70 rejected samples, reference results were available for 64 samples. Three were positive for *P. falciparum*, 11 were *P. vivax* and one was mixed infection. Since the device did not provide results for these samples, they could not be included in the analysis.

CL-IN002 was a single site clinical trial. Multi-site studies will help further validating the device. Beyond India, future tests sites will be placed throughout Africa, South East Asia as well as European areas demonstrating non-endemic malaria infections. A diversity of locations will further strengthen the device database and demonstrate the broad ability of the device to diagnosis a variety of genetic backgrounds.

## Conclusion

The SightDx P1 device demonstrated a sensitivity of 97.03 % and a specificity of 96.33 %, significantly exceeding the WHO criteria of 95 % accuracy and showing superior performance to RDT. Furthermore, since it can evaluate speciation and estimate the parasitaemia, it can be a valuable tool in clinical practice as well as research studies.
